# Immunogenicity of HLA Class I and II Double Restricted Influenza A-Derived Peptides

**DOI:** 10.1371/journal.pone.0145629

**Published:** 2016-01-05

**Authors:** Sara Ram Pedersen, Jan Pravsgaard Christensen, Søren Buus, Michael Rasmussen, Karen Smith Korsholm, Morten Nielsen, Mogens Helweg Claesson

**Affiliations:** 1 Institute of Immunology and Microbiology, Faculty of Health and Medical Sciences, University of Copenhagen, Copenhagen, Denmark; 2 Department of Infectious Disease Immunology, Statens Serum Institute, Copenhagen, Denmark; 3 Department of Systems Biology, Center for Biological Sequence Analysis, Technical University of Denmark, Lyngby, Denmark; 4 Instituto de Investigaciones Biotecnológicas, Universidad de San Martín, San Martín, Buenos Aires, Argentina; The University of Adelaide, AUSTRALIA

## Abstract

The aim of the present study was to identify influenza A-derived peptides which bind to both HLA class I and -II molecules and by immunization lead to both HLA class I and class II restricted immune responses. Eight influenza A-derived 9-11mer peptides with simultaneous binding to both HLA-A*02:01 and HLA-DRB1*01:01 molecules were identified by bioinformatics and biochemical technology. Immunization of transgenic HLA-A*02:01/HLA-DRB1*01:01 mice with four of these double binding peptides gave rise to both HLA class I and class II restricted responses by CD8 and CD4 T cells, respectively, whereas four of the double binding peptides did result in HLA-A*02:01 restricted responses only. According to their cytokine profile, the CD4 T cell responses were of the Th2 type. In influenza infected mice, we were unable to detect natural processing *in vivo* of the double restricted peptides and in line with this, peptide vaccination did not decrease virus titres in the lungs of intranasally influenza challenged mice. Our data show that HLA class I and class II double binding peptides can be identified by bioinformatics and biochemical technology. By immunization, double binding peptides can give rise to both HLA class I and class I restricted responses, a quality which might be of potential interest for peptide-based vaccine development.

## Introduction

HLA class I and II molecules are of importance for influenza virus processing and clearance. In primary influenza infections, cytotoxic, influenza-specific CD8 T cells are important for terminating the acute infection and they also contribute to long-term immunity [1,2]. CD4^+^ T cells may, in addition to their B cell supporting role in humoral immunity, act as direct effectors in protection against influenza and generate memory immunity [3].

We have previously identified 34 new HLA-class I binding influenza A-derived antigenic 9-mer peptides, which collectively bind to the twelve HLA class I supertypes [4,5], thus globally covering HLA by > 99%. Surprisingly, eighteen of these peptides gave rise exclusively to HLA class II restricted responses when assayed in ELISpot cultures using PBMC from immune donors ([2] and unpublished data). Similarly, 50% of PBMC from donors immunized with vaccinia virus and 100% of BCG vaccinated donors gave rise to HLA class II restricted responses when tested against HLA class I binding 9-mer peptides in IFNγ ELISpot assays [6,7].

We have speculated on the mechanisms behind presentation of such double restricted peptide epitopes, including the likelihood of autophagy and cross presentation or transmission of peptides from unstable HLA class I to class II molecules on the cell surface [8]. Functionally, immunization with peptides restricted by both HLA class I and II molecules might, due to increased peptide specific T helper activity, lead to higher levels of peptide specific CTL immunity in comparison with peptides restricted only by HLA class I molecules. The aim of the present study was to evaluate the immunogenicity of double restricted influenza-derived 9-11mer peptides in HLA-A*02:01/HLA-DRB1*01:01 transgenic mice. The HLA class I/II composition of our transgenic mouse model appears to be well warranted for studying the immunogenicity of double restricted influenza epitopes, first, because 50% and 20% of the European population carry the HLA-A*02:01 and the HLA-DRB1*01:01 allele, respectively [www.allelefrequencies.net], and secondly, because many CD8 T cell responses in influenza A virus infections appear to be restricted by the HLA-A*02:01 allele [9]. Furthermore, we also studied the protective effect of peptide immunization in mice challenged intranasally (i.n.) with influenza A virus.

## Materials and Methods

### Mice and Ethics

HLA-A*02:01/HLA-DRB1*01:01 transgenic H-2 class I-/class II-knockout mice on a C57BL/6 background were purchased from the Pasteur Institute, Paris, France [10]. Transgenic mice were bred and maintained in the animal facility of the Panum Institute, University of Copenhagen, Denmark, and all mice were at least 7 weeks old before entering into experiments. Mice were housed under controlled microbial conditions. Experiments were conducted in accordance with national Danish guidelines (Amendment # 1306 of November 23, 2007) regarding animal experiments as approved by the Danish Animal Experiments Inspectorate, Ministry of Justice, permission number 2009/561-1679.

### Bioinformatics search strategy for CTL and Th epitopes derived from influenza A virus

Epitope predictions were performed on the basis of datasets obtained from the Influenza Sequence Database (www.flu.land.gov). Only peptides from influenza A-derived constant proteins such as M1, M2, NS1, PB1, PB2, PA and NP proteins were included in the analysis, while the variable proteins N and H were excluded. Bioinformatic analyses were used to identify double binding influenza A-derived peptides, i.e. peptides predicted to bind to both HLA-A*02:01 and to HLA-DRB1*01:01. Binding to HLA-A*02:01 was performed using NetMHCpan-2.8 [11,12]. Binding to HLA-DRB1*01:01 was performed using NetMHCIIpan-3.0 [13,14] and both prediction methods are available at www.cbs.dtu.dk/services. Analyses were performed using the Influenza strain A/Puerto Rico/8/1934(H1N1) [Refseq accession numbers:NP_040978.1,NP_040979.2,NP_040980.1,NP_040981.1,NP_040982.1,NP_040983.1,NP_040984.1,NP_040985.1,NP_040986.1,NP_040987.1].

Peptides were selected based on the following strategy. First peptides of length 9–11 amino acids with predicted binding to HLA-A*02:01 stronger than 50nM were selected. Next this list was filtered to include only peptides that either were predicted to bind HLA-DRB1*01:01 or were part of a 15mer peptides that was predicted to bind HLA-DRB1*01:01 with an affinity stronger than rank 10% (the IEDB recommended threshold for MHC class II peptide binding classification). Finally, the selection was extended by a peptide that is part of a 15mer with very strong binding to HLA-DRB1*01:01 even though it lacked binding to HLA-A*02:01, and a peptide with strong binding to HLA-A*02:01 even though it lacked binding to HLA-DRB1*01:01 (details on the peptide selection and predicted binding values are given Table 1). These peptides were all present in the influenza strain A/Puerto Rico/8/34 H1N1 used for the challenge of mice.

**Table 1:** Peptides predicted and measured for binding to HLA-A*02:01 and HLA-DRB1*01:01.

Protein	Peptide sequence	Response	HLA-A*02:01		HLA-DRB1*01:01		HLA-DRB1*01:01 15mer	
			Measured nM	Predicted nM	Measured nM	Predicted %rank	Peptide sequence	Predicted %rank
PA(282–292)	FLLMDALKLSI	I/II*	24	15.63	23	0.05		
PB1(410–419)	GMFNMLSTVL	I/II	384	35.1	29	5		
PA(282–290)	FLLMDALKL	I/II	14	15.42	46	0.1		
PB1(408–418)	MMGMFNMLSTV	I/-	51	10.59	172	5		
PB1(318–326)	FLAMITYMT	I/-	253	17.03	557	1.5		
NS2(99–107)	FMQALHLLL	I/-	13	9.74	>5000	5		
NP(258–266)	FLARSALIL	-/-	0.9	61.5	97	8		
PB2(49–57)	WMMAMKYPI	-/-	36	3.75	>5000	7		
PB2(49–59)	WMMAMKYPITA	-/-	75	12.31	528	3		
M(41–51)	VLMEWLKTRPI	-/-	101	9.71	119	9		
PB1(407–417)	MMMGMFNMLST	-/-	36	27.25	315	5		
PB1(217–227)	YLIRALTLNTM	-/-	46	28.42	76	1.5		
NP(297–306)	SLVGIDPFRL	-/-	70	43.31	460	6		
PB1(410–418)	GMFNMLSTV	I/II	23	6.85	34	32	GMFNMLSTVLGVS	2
NS1(128–136)	IILKANFSV	I/-	45	19.35	>5000	15	KNIILKANFSVIF	3
M(134–142)	RMGAVTTEV	I/-	133	28.24	>5000	50	LIYNRMGAVTTEV	0.8
M(58–66)	GILGFVFTL	I***/-	3	15.03	>5000	50	GILGFVFTLTVPSER	5
PB1(413–421)	NMLSTVLGV	-/-	16	9.4	>5000	32	GMFNMLSTVLGVS	2
NP(306–315)	LLQNSQVYS	(I)/-	1369	3168.34	>5000	50	PFRLLQNSQVYSL	0.1
PB1(166–174)	FLKDVMESM	I**/-	51	23.43	>5000	32		
Reference peptide	YKYVKQNTLKLATHHHHHH		-		8			

**Fig 1:**
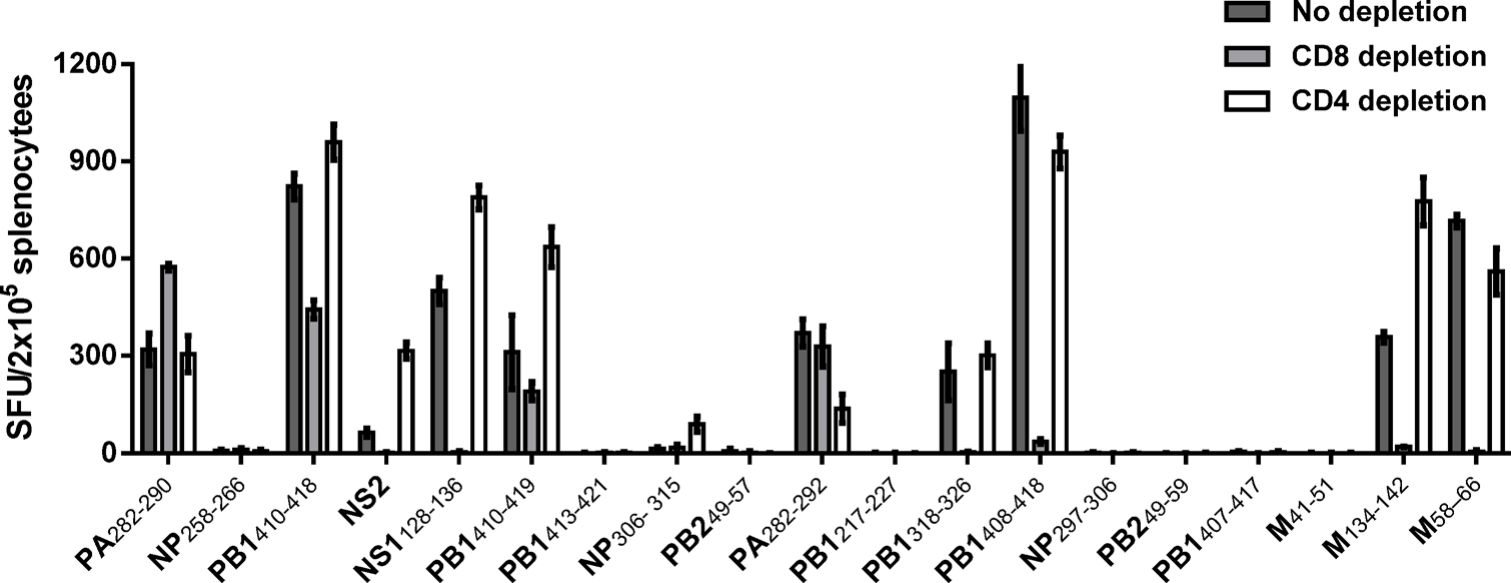
Induction of double restricted responses after peptide immunization of HLA transgenic mice. Eighteen groups of 3–5 mice were injected intraperitoneally three times at two week intervals with CAF09 plus one of the 9-11mer peptides (20nM). One week after the last immunization, IFNγ ELISpot was used to measure HLA-A*02:01 and/or HLA-DRB1*01:01 restricted CD8 and CD4 T cell responses. As a control, a group of mice (n = 4) was injected intraperitoneally three times at two week intervals with CAF09 plus the known HLA-A*02:01 binding peptide M(58–66) (21). Each bar represents mean ± SD of IFNγ spot forming units (SFU) of groups of 3–5 immunized mice from which splenocytes were pooled and depleted for CD4 or CD8 positive cells before being stimulated *ex vivo* with peptide in four replicate cultures. Experiments were conducted 1–5 times depending on the individual peptides.

*Type of immune response: I, CD8 response; II, CD4 response; -,no response (conf. Fig 1)

**Known HLA-A*02:01 binding and processable influenza peptide [21].

***Measured by FACS (conf. Table 2).

### Peptides

The peptides were synthesized at Schafer N, Copenhagen, Denmark or at Zhengzhou Peptides Pharmaceutical technology Co., Ltd. Zhengzhou City, Henan Province P.R China by standard 9-fluorenylmethyloxycarbonyl (FMOC) chemistry, and purified by reversed-phase high-performance liquid chromatography (at least 80%, usually >95% purity) and validated by mass spectrometry (Shafer-N, Copenhagen, Denmark). Peptides were dissolved before each experiment.

### Biochemical peptide-HLA-I and–II binding assays

The biochemical assay for peptide–MHC-I binding was performed as previously described [15,16]. Briefly, denatured and purified recombinant HLA heavy chains were diluted into a renaturation buffer containing β_2_-microglobulin and graded concentrations of the test peptide, and incubated at 18°C for 48h allowing equilibrium to be reached. We have previously demonstrated that denatured HLA molecules can *de novo* fold efficiently, however, only in the presence of appropriate peptide [12]. The concentration of peptide–HLA complexes generated was measured using Luminescent Oxygen Channeling Immunoassay (LOCI) and plotted against the concentration of peptide offered. Because the effective concentration of HLA (in casu 0.5nM) used in these assays is below the equilibrium dissociation constant (K_D_) of most high-affinity peptide–HLA interactions, the peptide concentration leading to half-saturation of the HLA is a reasonable approximation of the affinity of the interaction.

Affinity measurements of peptides to recombinant HLA-DRA*01:01/DRB1*01:01 molecules were done according to previous work with minor modifications [17]. Briefly, peptides including a reference peptide, known to bind to HLA-II DR (HA 308–318, YKYVKQNTLKLAT) [18], were dissolved and titrated in 25% glycerol, 0.1% pluriol (F68), PBS. An HLA-II stock solution consisting of bacterially expressed and urea denatured alpha and beta chains at appropriate concentrations was diluted into refolding buffer (100 mM Tris/Citrate, 25% Glycerol, 0.01% Pluriol, pH 7). The diluted HLA-II stock was subsequently mixed 1:1 with peptide titrations and incubated at 18°C for 48h. Formed HLA-II complexes were detected using a homogenous proximity assay (Alphascreen, Perkin Elmer, USA), briefly streptavidin coated donor beads and in-house L243 (murine monoclonal anti-DR) conjugated acceptor beads, both 5 mg/ml, were diluted into PBS 0.1% Pluriol F68 and added to the HLA-II complexes giving a final concentration of 5μg/ml of each. Following 18h of incubation at 18°C they were read on an EnVision Reader (Perkin Elmer, USA) and analyzed according to [17].

### Immunization of mice

Peptides were dissolved and mixed with Freund’s Incomplete Adjuvant (FIA) (Sigma, USA), Freund’s Complete Adjuvant (FCA) or CAF09 (Statens Serum Institute, Denmark). CAF09 is a novel cationic liposomal adjuvant formulation comprised of DDA (dimethyldioctadecylammonium, 250 μg/dose) liposomes combined with MMG-1 (monomycoloyl glycerol 1, 50 μg/dose) and Poly(I:C) (50 μg/dose) [19]. Mice were immunized two or three times with either 20 or 50 nmol peptide at two week intervals. When using Freunds’s Adjuvants, peptides were dissolved in PBS, mixed with equal amounts of adjuvant and 100 μl were administered subcutaneously (s.c.). Peptides given with CAF09 were dissolved in 10 mM Tris-buffer, mixed with equal amounts of CAF09 and injected intraperitoneally (i.p.) in 200 μl.

### ELISpot assay

96 well Multiscreen HTS ELISpot plates (Millipore, Germany) were coated overnight at 4°C with unlabeled anti-mouse Ab purchased at BD Bioscience, USA (3 μg/ml for anti-IFNγ or 5 μg/ml for anti-IL-4). The next day, plates were washed 6 times with blocking solution (RPMI 1640 supplemented with 10% FBS and 1% penicillin, streptomycin and L-glutamine). 200 μl blocking solution was added and plates incubated for at least 2h at room temperature. Cells were prepared by pressing spleens trough a fine steel mesh, and splenocytes from all mice in a group were subsequently pooled. Cells were then added to wells (2x10^5^ cells/well for IFNγ assays and 1x10^6^ cells/well for IL-4 assays) and peptides were added in a final concentration of 20 nmol/ml. For non-depleted samples, 2x10^5^ or 1x10^6^ cells/well were added directly from the pooled cell population. For CD4 or CD8 depleted samples, a fraction of the pooled cells was first depleted for CD4^+^ or CD8^+^ cells (described in detail below) before 2x10^5^ or 1x10^6^ of the depleted cells were added to wells. Each sample was run in four replicates. Concanavalin A (ConA) stimulated cells (3 μg/ml) were included as a positive control and unstimulated cells were used as a negative control. Plates were incubated for 18-22h for IFNγ assays and 40-44h for IL-4 assays at 37°C and 5% CO_2_. Plates were washed twice with Milli Q H_2_O, allowing wells to soak 3–5 min at each step. Next, plates were washed four times with washing buffer (PBS containing 0.005% Tween 20), letting wells soak for 1–2 min at each step. Biotinylated Ab (BD Biosciences, USA) was diluted in PBS with 1% BSA to a final concentration of 1.2 μg/ml (anti-IFNγ) or 2 μg/ml (anti-IL-4) and 100 μl was added to wells. Plates incubated at room temperature for 2h. Plates were then washed 6 times with washing buffer, while allowing wells to soak 1–2 min at each washing step. HRP Streptavidin (BD Bioscience, USA) was diluted 1:100 in PBS with 10% FBS and 100 μl was added to each well. Plates incubated for 1h at room temperature. HRP Streptavidin was discarded and the protective plastic bottom was removed from the plates. Next, wells were washed 6 times with washing buffer and twice with PBS, allowing plates to soak for 1–2 min at each step. 100 μl of EAC substrate reagent set (BD Bioscience, USA) was next added to each well and spots were allowed to develop (30–60 sec). The AEC was discarded appropriately and the reaction was stopped by the addition of Milli Q H_2_O to the wells. Finally, plates were washed 6 times with Milli Q H_2_O and dried at room temperature in the dark for 1 or more days before spot forming units were counted using a CTL-Immunospot S6 Macro Analyzer (CTL, Germany).

### Spleen cell preparation and intracellular cytokine measurements

Spleens were isolated and single cell suspensions were obtained by pressing the organs trough a fine steel mesh; subsequent staining for flow cytometry was performed as previously described [20]. For intracellular staining, 2x10^6^ splenocytes were re-suspended in supplemented RPMI 1640 and stimulated *ex vivo* with relevant peptides in the presence of IL-2 (50 IU/ml) and monensin (2 μg/ml; to block Golgi vesicle transport). Control cultures did not receive any peptide. After 5 hours of stimulation, cells were stained for cell surface markers, followed by permeabilization and intracellular staining as described [20]. Cells were analyzed using a FACS Calibur (BD Biosciences, USA). At least 10^4^ mononuclear cells were gated using a combination of side and forward scatter to exclude dead cells and debris. Data analysis was conducted using FlowJo (Tree Star, USA). The gating strategy for FACS analysis is shown in the supplementary material, S1 Fig.

### CD8^+^ and CD4^+^ T cell depletion

Spleens, bronchoalveolar fluids and mediastinal lymph nodes were isolated and single cell suspensions were obtained by pressing the organs trough a fine steel mesh. Cells were mixed with either rat anti-CD4 or anti-CD8 Ab (clone YTS191 or YTS169, a kind gift by Steven Cobbold, University of Oxford) and left on ice for 30 min. Sheep anti-Rat IgG Dynabeads (Life Technologies, USA) (100μl/2x10^7^ cells) were washed and used for depletion. After cells had incubated with Ab for 30 min, they were washed twice with 5 ml RPMI supplemented with 10% FBS, 1% L-glutamine, penicillin and streptomycin, as well as 2-ME (5x10^-5^ Mol). Cells were resuspended in 5 ml supplemented RPMI and Dynabeads were added. Tubes were placed in an end-over-end shaker for 40 min at 4°C. Finally, tubes were placed on a magnet and depleted cell suspensions were isolated and washed once in supplemented in RPMI before being added to the ELISpot plates. As a control, surface staining for CD8 and CD4 was completed after each experiment using flow cytometry to ensure that depletion had been satisfactory. The results of a representative cell depletion experiment are shown in the supplementary material, S2 Fig.

### Antibodies for flow cytometry

The following mAb were used as rat anti-mouse antibodies: PerCP-Cy5.5-conjungated anti-CD8, FITC-conjungated anti-CD44, PE-conjungated anti-CD4, APC-conjungated anti-IFNγ (Nordic Biosite, Sweden).

### Influenza strains and influenza challenge

For evaluation of clinical relevance, mice were challenged with Influenza A/Puerto Rico/8/34 (H1N1). Briefly, the mice were anaesthetized by i.p. injection of 300–500 μl of Avertin (25 mg/ml) and infected i.n. with graded doses of virus and the LD_50_ was estimated. The weight of the mice was monitored daily and mice suffering from a weight loss greater than 25% of their starting weight were sacrificed for humane reasons. In challenge experiments, mice were immunized i.p. three times and then challenged with 4, 0.4 or 0.04 LD_50_ of influenza one week after the final immunization. Lungs were then isolated 5 days post infection and snap frozen in liquid nitrogen. Subsequently, lungs were stored at -80°C before being tested for levels of virus by Q-PCR (see below). For peptide response experiments, mice were challenged with 4 LD_50_ or 0.4 LD_50_ of influenza and 10 days later, spleens, bronchoalveolar fluids, and mediastinal lymph nodes were collected and tested for peptide reactivity in IFNγ ELISpot or intracellular FACS staining.

### RNA purification

Mice were euthanized and lungs were isolated and immediately snap frozen in liquid nitrogen. Total RNA was extracted using an RNeasy midi kit (Qiagen, Hilden, Germany) as described by the manufacturer. Briefly, lungs were homogenized in a lysis buffer using a homogenizer (Bie & Berntsen, Denmark), cell suspensions were centrifuged and supernatants were isolated and mixed with 70% ethanol. Lysates were added to RNeasy midi spin columns and spun, to bind the RNA to the filters. After several washing steps, total RNA was eluted from the column by the addition of RNase free water. Finally, the total RNA concentration and purity was measured using a NanoDrop 2000c Spectrophotometer (Thermo Fischer Scientific, USA) and the associated computer program NanoDrop 2000.

### Quantitative real time polymerase chain reaction (Q-PCR)

One-step duplex Q-PCR was performed with 0.1 μg total RNA per sample using a 1-step Brillant II Q-RT-PCR Master Kit (Stratagene, USA). The Q-PCR program was: 30 min/50°C, 10 min/95°C; followed by 40 cycles of 30 sec/95°C, 1 min/58°C, and 30 sec/72°C. The following primers and probes were used: M probe: 5’ FAM—TCA GGC CCC CTC AAA GCC GA—BHQ ‐ 1 3’, M forward primer: 5’ AGA TGA GTC TTC TAA CCG AGG TCG 3’, M reverse primer: 5’ TGC AAA AAC ATC TTC AAG TCT CTG 3’, GAPDH probe: 5’ HEX—CGC CTG GAG AAA CCT GCC AAG TAT—BHQ – 1 3’, GAPDH forward primer: 5’ CAA TGT GTC CGT CGT GGA 3’, GAPDH reverse primer: 5’ GAT GCC TGC TTC ACC ACC 3’. Each sample was run in triplicates and a no template control was added in duplicates to detect any possible background signals. To evaluate the efficiency of the amplification, a -10x sample titration series was added in duplicates. Also, a -10x plasmid titration series of a paCCMV plasmid encoding the coding region of the influenza M gene was added in duplicates. This plasmid titration curve was used for absolute quantifications of the total number of copies of the influenza M gene in the organ samples. The Q-PCR data analysis was performed on a Mx3005P Real-time Q-PCR instrument, and the results were analysed using the software programme MxPro–Mx3005p v. 4.1 (Stratagene, USA).

### Statistical analysis

Quantitative results were compared using the Mann–Whitney *U* test, and *p* values < 0.05 were considered statistically significant.

## Results

### Prediction and biochemical validation of 9-11mer influenza epitopes binding to HLA-A*02:01 and HLA-DRB1*01:01

Initially, we were interested in identifying 9-11mer epitopes from conserved proteins (M1, M2, NS1, PB1, PB2, PA and NP proteins) derived from the H1N1 influenza strain A/Puerto Rico/8/34 capable of binding to both HLA-A*02:01 and HLA-DRB1*01:01. Epitope predictions were performed (see M&M section) and nineteen 9-11mer peptides predicted to bind to HLA-DRB1*01:01 and/or HLA-A*02:01 were identified. These peptides were synthesized and tested for binding to recombinant HLA-A*02:01 and HLA-DRB1*01:01 molecules in a biochemical system (see M&M section). Table 1 shows the results of these binding assays. The overall agreement between the predicted and measured binding values is high. 15 peptides were measured to bind to HLA-A*02:01 with high affinity (KD < 100 nM), and only one peptide (the one that lacked predicted binding) failed to bind HLA-A*02:01.Eight of the peptides were measured to bind to HLA-DRB1*01:01 with a KD value below 200 nM. These all corresponded to peptides with a predicted rank < 10% (the IEDB recommended threshold for MHC class II peptide binding classification). Only one of the peptides selected as nested binders within a 15mer peptide showed binding to DRB1*01:01, which is in general accordance with the predicted binding values. Seven of the HLA-DRB1*01:01 binding peptides also showed high affinity for HLA-A*02:01. Thus, it appears that a significant number of 9-11mer influenza-derived peptides can bind simultaneously to both HLA class I and II molecules. Table 1 includes a HLA-DRB1*01:01 19mer reference peptide [18] and the known HLA-A*02:01 binding influenza matrix M(58–66) peptide [21].

The immunogenic properties of these double binding peptides were further tested in a transgenic mouse model.

### Cationic adjuvant formulation 09 (CAF09) induces strong T cell responses

To investigate the *in vivo* immunogenicity of the peptides depicted in Table 1, we had to determine an appropriate immunization platform. Since Freund’s adjuvants have been widely used for peptide immunization in the literature, including studies done with influenza peptides in our group, we considered it a possible candidate. Table 2 includes immunization data obtained from 13 groups of 2–3 mice each. Initially, we determined the percentage of IFNγ producing splenic CD8 and CD4 T cells from HLA transgenic mice immunized s.c. at different time points, with different combinations of FCA or FIA, and in combination with different amounts of influenza peptide PA (282–290), PB1(410–418) and NS1(128–136). These peptides showed strong to intermediate binding affinities for both class I and II molecules (Table 1). The known HLA-A2 binding and processable peptide M(58–66) [21] was included as a positive control for a HLA-A2 restricted response. Intracellular staining revealed that no marked IFNγ production was detected from either CD8 or CD4 T cells when immunizing with FCA and/or FIA. A minor CD8 T cell response was, however, detected for NS1(128–136) and M(55–66) in two different setups. On the other hand, when immunizing HLA transgenic mice i.p. with peptides combined with the recently developed cationic liposome adjuvant CAF09 (15), high levels of IFNγ was produced by CD8 T cells specific for influenza peptides PA(282–290), PB1(410–418) as well as M(58–66). Thus, three biweekly injections with 20 nmol peptide and CAF09 as an adjuvant were therefore used as an immunization platform for the remainder of the study.

**Table 2:** FACS analysis of splenic T cells*.

Adjuvant	Immunization peptide(s)	Amount of peptide	Days of immun.	Peptide for stimulation	% IFNγ prod.CD8 cells	% IFNγ prod.CD4 cells
**FCA/FIA**	PB1(166–174) + NS1(128136)	20 nmol	0 and 14	PB1(166–174)	0.037 ± 0.035	0
**FCA/FIA**	PB1(166–174) + NS1(128–136)	20 nmol	0 and 14	NS1(128–136)	1.267 ± 1.462	0
**FIA**	PB1(166–174) + NS1(128–136)	50 nmol	0 and 14	PB1(166–174)	0	0.003 ± 0.003
**FIA**	PB1(166–174) + NS1(128–136)	50 nmol	0 and 14	NS1(128–136)	0.211 ± 0.082	0.007 ± 0.008
**FIA**	PB1(166–174) + NS1(128–136)	50 nmol	0 and 21	PB1(166–174)	0.003 ± 0.006	0.003 ± 0.006
**FIA**	PB1(166–174) + NS1(128–136)	50 nmol	0 and 21	NS1(128–136)	0.200 ± 0.157	0.007 ± 0.006
**FIA**	PA(282–290)	20 nmol	0 and 14	PA(282–290)	0.270 ± 0.311	0
**FIA**	M(58–66)	20 nmol	0 and 14	M(58–66)	0.285 ± 0.361	0
**FIA**	PA(282–290)	20 nmol	0, 14 and 28	PA(282–290)	0.447 ± 0.285	0.07 ± 0.012
**FIA**	PB1(410–418)	20 nmol	0, 14 and 28	PB1(410–418)	0.643 ± 0.860	0.003 ± 0.006
**FIA**	NP(258–266)	20 nmol	0, 14 and 28	NP(258–266)	0.017 ± 0.029	0
**FIA**	M(58–66)	20 nmol	0, 14 and 28	M(58–66)	2.487 ± 0.496	0.003 ± 0.006
**CAF09**	PA(282–290)	20 nmol	0, 14 and 28	PA(282–290)	14.457 ± 6.160	0.440 ± 0.442
**CAF09**	PB1(410–418)	20 nmol	0, 14 and 28	PB1(410–418)	18.557 ± 13.515	0.013 ± 0.015
**CAF09**	NP(258–266)	20 nmol	0, 14 and 28	NP(258–266)	0.040 ± 0.061	0.007 ± 0.006
**CAF09**	M(58–66)	20 nmol	0, 14 and 28	M(58–66)	15.157 ± 1.,019	0.023 ± 0.040

* Subcutaneous and intraperitoneal immunization approaches with different HLA-A*02:01 and DRB1*01:01 binding peptides in HLA transgenic mice (n = 2–3). Each mouse was evaluated individually by intracellular IFNγ staining and FACS analysis of splenic T cells and cells analyzed were gated as CD8^+^IFNγ^+^ or CD4^+^IFNγ^+^. All immunizations using Freund’s adjuvants (FCA or FIA) were given subcutaneously, while CAF09 was administered by intraperitoneal injections. The experiments were conducted once.

### Four influenza A-derived epitopes show HLA class I/II double restricted responses in IFNγ ELISpot measurements

Having decided on an immunization approach, we next investigated which, if any, of the predicted and validated influenza A epitopes shown in Table 1 could in fact induce HLA-I and–II double restricted T cell responses. Hence, we immunized 72 transgenic mice in groups of 3–5 animals with the individual 9-11mer peptides and CAF09 as adjuvant and examined the epitope-specific T cell responses one week after the last immunization. Mice immunized with M(58–66) were included in all experiments as a positive control. Mice immunized with M(58–66) were included in all experiments as a positive control. Since preliminary results had found ELISpot assay to display more than 10 times higher sensitivity and a lower background than intracellular IFNγ staining and FACS analysis (S1 Fig), we now used this technology to determine responses in unfractionated T lymphocytes and cells depleted for CD8 or CD4 positive cells. Fig 1 shows the immunization data which are also summarized in Table 1 (left column). We examined a total of 19 peptides and observed 8 peptides, including four HLA-A*02:01/DRB1*01:01 double bindings ones, which were not immunogenic at all. Seven epitopes were found to be exclusively HLA-A*02:01 restricted, including the known HLA-A*02:01 epitope M(58–66) [21] whereas no peptide appeared to be only HLA-DRB1*01:01 restricted. Finally and most important, four 9-11mer peptides; PA(282–290), PA(282–292), PB(410–418) and PB1(410–419), were observed to elicit both CD8 and CD4 T cell responses.

**Fig 2:**
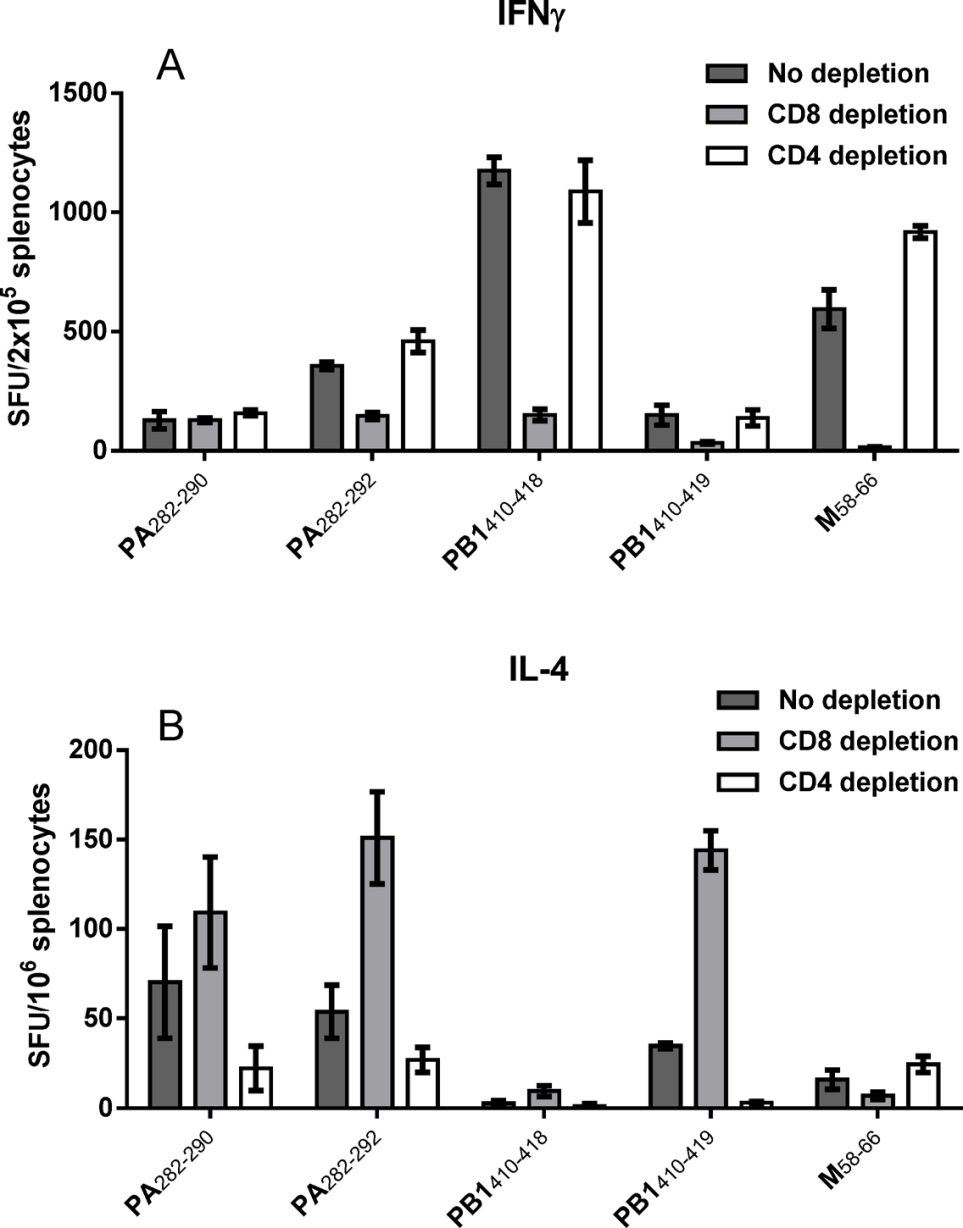
Immunization with the four double restricted epitopes induces both CTL and Th2 reactivity. Five groups of HLA transgenic mice were immunized intraperitoneally three times at two week intervals with CAF09 plus one of the four double restricted peptides or M(58–66) (20nM) [21]. One week after last immunization, IFNγ (A) or IL-4 (B) ELISpot was used to measure spot forming units (SFU) from splenocytes. Cells were pooled from each group and depleted for CD4 or CD8 positive cells before being stimulated *ex vivo* with peptide in four replicate cultures. Each bar represents mean ± SD of IFNγ (A) or IL-4 (B) spot forming units (SFU) of groups of 4 immunized mice. Experiments were conducted twice.

To examine if the double restricted responses primarily induces a Th1 or Th2 response, we looked into the expression of the Th2 cytokine IL-4 as well as the Th1 cytokine IFNγ after immunization of individual mice with the four double restricted peptides and M(58–66). As shown in Fig 2, T cells depleted of CD8 cells showed high levels of IL-4 secretion and low levels of IFNγ production indicating their Th2 nature, whereas CD4 depleted cell populations as expected showed high levels of IFNγ production typically for activated CTLs. As expected, immunization with the HLA-A*02:01 restricted peptide M(58–66) [17]only gave rise to a CTL response. These results show that immunization with three of the four double restricted epitopes found in this study will induce both CTL and Th2 reactivity.

### Lack of natural responses against the influenza-derived peptides

**Fig 3:**
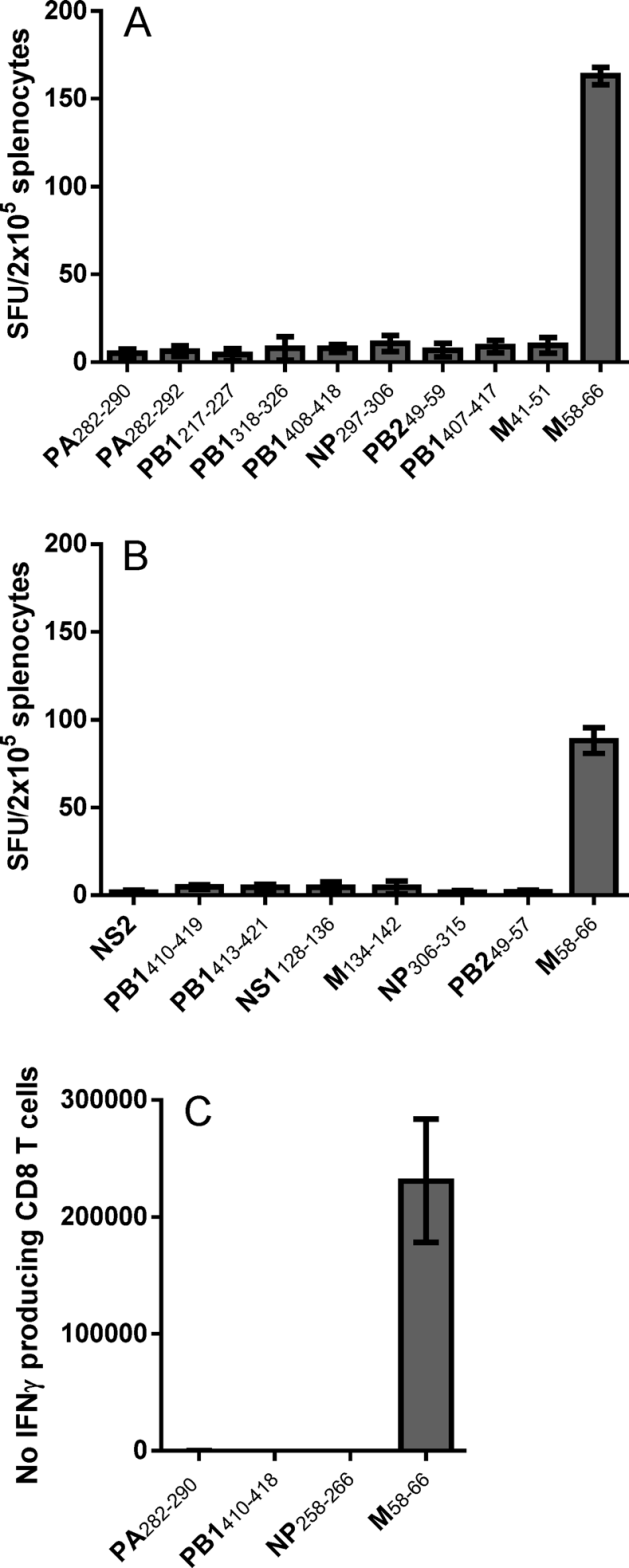
Influenza infected HLA transgenic mice show no reactivity towards any of the immunogenic peptides. HLA transgenic mice were challenged intranasally with 4 LD_50_ (B) or 0.4 LD_50_ (A and C) of influenza A. IFNγ production from splenocytes was detected 10 days later using IFNγ ELISpot (A and B) or flow cytometry (C). In A and B, each bar represents mean ± SD of IFNγ spot forming units (SFU) of groups of 4–5 mice from which splenocytes were pooled before being stimulated *ex vivo* with peptide in four replicate cultures. In C, each bar represents mean ± SD of the number of IFNγ producing splenic CD8 T cells from 2 individual mice after *ex vivo* stimulation with peptide. Experiments were conducted 1–4 times depending of the peptide. Experiments were conducted 1–4 times, depending on the peptide.

In order to determine if the peptides used in this study, including the four immunogenic, double restricted epitopes, are processed and presented to the immune system during natural infection, we next investigated peptide specific responses in influenza (PR8) challenged HLA transgenic mice. Fig 3 shows data from individual influenza challenge experiments (2–5 mice per experiment) in which splenic T cells from influenza PR8 infected mice were tested in IFNγELISpot for recognition of the influenza peptides depicted in Table 1 and Fig 1. Included is the known HLA-A*02:01 binding M(58–66) peptide [21] as a known positive control. As shown in Fig 3, reactivity was only observed against peptide M(58–66), suggesting that only this peptide is naturally processed during viral infection. Furthermore, this lack of response against epitopes, of which the majority was predicted and measured to bind to HLA-A*02:01 with high affinity, was also evident in T cells isolated from both bronchoalveolar lavage fluids and mediastinal lymph nodes from influenza challenged mice (data not shown).

### Virus clearance in influenza infected mice immunized with double restricted peptides

**Fig 4:**
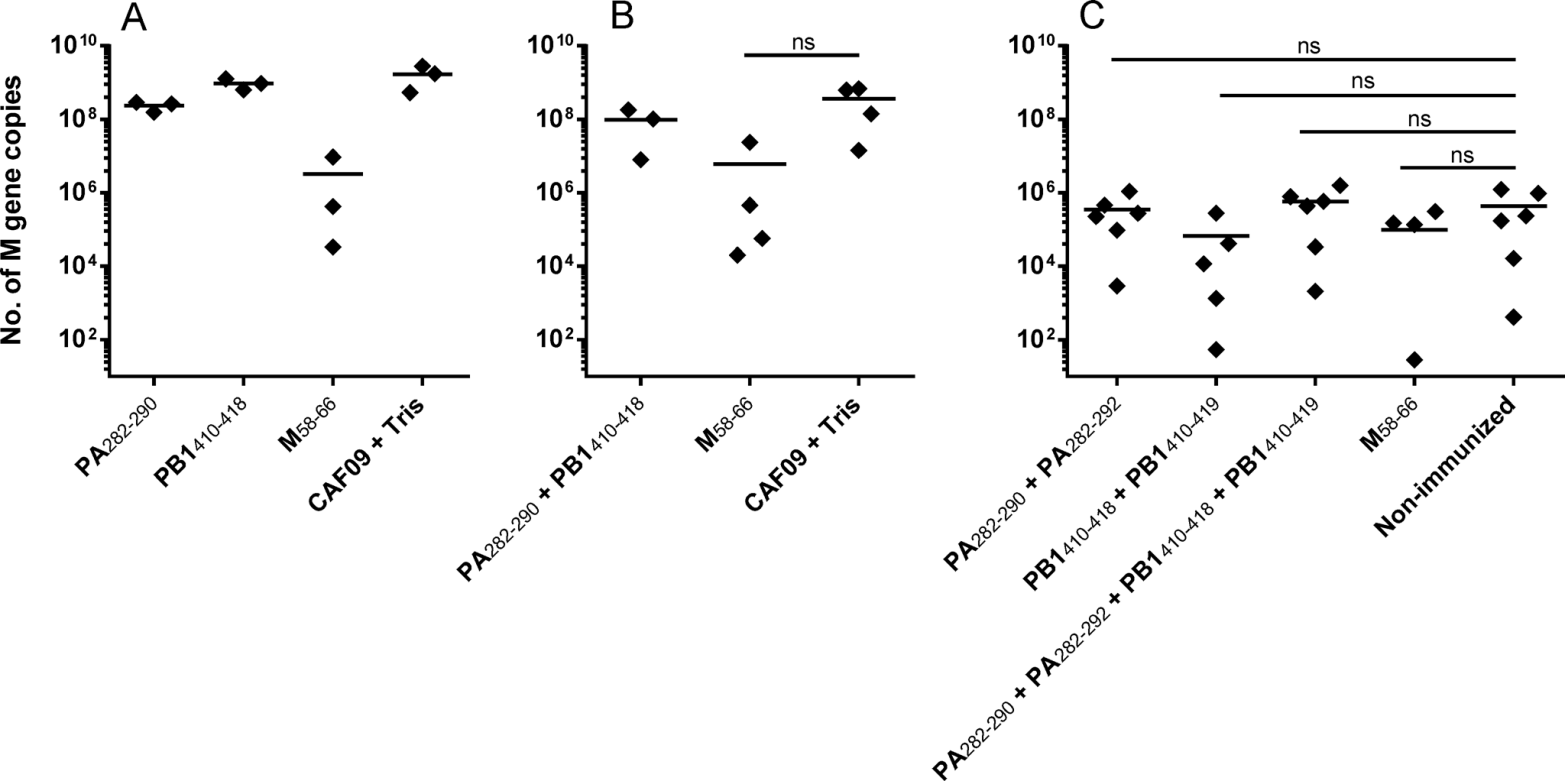
No protection against influenza infection in mice immunized with the double restricted epitopes. HLA transgenic mice were immunized three times every second week with CAF09 and different combinations of double restricted immunogenic peptides or the known HLA-A*02:01 epitope M(58–66) (20nM). One week post last immunization, mice were intranasally challenged with 4 LD_50_ (A), 0.4 LD_50_ (B), or 0.04 LD_50_ (C) of influenza A. As control groups, mice were either injected three times at two week intervals with CAF09 and Tris buffer (A and B) or were non-immunized (C) before influenza challenge. Influenza M gene expression in the lungs was measured by Q-PCR. Dots show numbers of M gene copies in the lungs per mice and horizontal lines depict means of 3–6 mice. Experiments were conducted once. Groups were tested using Mann-Whitney and ns = not significant compared to CAF09 + Tris (B) or non-immunized (C). Groups in A could not be tested by Mann-Whitney due to n = 3 in each group. Experiments were conducted once. Groups were tested using Mann-Whitney and ns = non significant compared to CAF09 + Tris (B) or non-immunized (C). Groups in A could not be tested by Mann-Whitney due to n = 3 in each group.

In spite of the apparent lack of immune reactivity against the four HLA-A*02:01/HLA-DRB1*01:01 binding immunogenic peptides in influenza challenged mice, we speculated if T cells from immunized mice with specificity for the double restricted peptides (Figs 1 and 2), might still be capable of killing directly infected cells and thereby lower the virus titre. Thus, groups of 3–6 transgenic mice were immunized three times with one or a combination of PA(282–290, PA(282–292), PB1(410–418) and PB(410–419. Separate groups of mice were immunized with the HLA-A*02:01 binding M(58–66) peptide [21] and groups of mice were left unimmunized or treated with CAF09 and Tris buffer. One week after the last immunization, mice were challenged i.n. with 4.0, 0.4 and 0.04 LD_50_ of influenza A and 5 days later, influenza M gene expression in lungs was measured by Q-PCR. Fig 4 shows that mice immunized with the M(58–66) peptide and challenged with influenza A at 4.0 and 0.4 LD_50_ showed reduced virus load, whereas immunization with the PA and PB1 peptides did not show any reduction in virus load irrespectively of the influenza dose used for challenge.

## Discussion

We have previously identified CD8 and CD4 T cell reactivity towards HLA class I and class II double binding immunogenic 9mer influenza A-derived peptides in the peripheral blood of healthy adult blood donors. Double binding or double restricted peptides were present for all the twelve HLA class I supertypes [5]. T cell reactivity against HLA class I/II double binding immunogenic 9mer peptides was also observed in the peripheral blood of donors vaccinated 20–40 years previously against smallpox and/or tuberculosis [6,7] indicating that reactivity against double binding infection- or vaccination-derived peptides reflects the presence of long term T cell memory. Intuitively, a peptide derived from an infectious agent triggering both HLA class I and class II restricted T cell responses against the same epitope might induce higher levels of immune protection than any peptide separately triggering a HLA class I or a class II restricted response, respectively [8]. To further address this hypothesis, we here focused on influenza A-derived peptides predicted *in silico* to bind to both HLA-A*02:01 and HLA-DRB1*01:01 molecules. After peptide synthesis, their binding to HLA-A*02:01 and HLA-DRB1*01:01 molecules was measured in a biochemical assay system [17]. The overall agreement between the predicted and measured binding values of the 19 synthesized peptides was high (see Table 1). The peptides were then tested for immunogenicity in HLA-A*02:01/HLA- DRB1*01:01 transgenic mice. Of the eight peptides measured to bind to both HLA-A*02:01/HLA-DRB1*01:01, four peptides showed HLA class I/class II double restricted responses and the HLA class II restricted responses represented in particular Th2 (IL-4) reactivity. These four peptides included two 9mer epitopes and their two extended versions, elongated with either one or two amino acids. Ten peptides binding to HLA-A*02:01 showed HLA-A*02:01 restricted responses whereas eight peptides, in spite of high HLA-A*02:01 binding affinities, were not immunogenic in these experiments. We had expected to find that splenic T cells from mice, challenged with Influenza A/Puerto Rico/8/34, would display reactivity towards at least some of the 18 assayed peptides and in particular the four immunogenic, double restricted peptides. However, reactivity was only observed against the influenza matrix M(58–66) peptide, the resulting CTL response known to be detectable in most HLA-A*02:01 influenza experienced subjects [17]. Nonetheless, the present results agree quite well with previous data [5,7,22] in which only 5–10% of more than 600 9mer peptides derived from influenza A virus, small pox virus, or mycobacterium tuberculosis were recognized by peripheral blood T cells of immune donors, although these peptides were predicted and measured biochemically for binding at high affinities to one or more of the twelve HLA class I supertypes [4,5,7,22].The reason for low numbers of immunogenic or immune dominant peptides in a given antigenic molecule is probably caused by limitations in the CD8 T cell repertoire as well as inefficient antigen processing which, according to previous studies, appears to reduce the number of immunogenic peptide epitopes within a given antigenic molecule by more than 90% [23]. Also, throughout evolution, influenza A virus might, in a process of host adaption, have been able to eliminate strong immunogenic peptides from their genome including double HLA-I/II binding peptides. In addition, in naïve HLA-A*02:01/HLA-DRB1*01:01 transgenic mice, CD8 T cells represent only 2–3% of the peripheral T cell pool [24] and might therefore reduce the overall CD8 T cell repertoire in these mice.

Unlike in the afferent arm of the immune system, peptide specific effector CD8 T cells (CTL) need to recognize only a few MHC/peptide complexes to become activated and kill [25]. Thus, although splenic T cells from influenza challenged mice did not recognize the HLA double binding peptides, or any of the other 19 HLA binding peptides of this study, predicted to be single or double restricted, we speculated that directly influenza infected target cells might process peptides at a level high enough to trigger low numbers of vaccine-induced CTLs not detectable in influenza challenged mice by using the ELISpot system. To test this possibility, peptide vaccinated and naive mice were challenged i.n. with titrated numbers of the Puerto Rico/8/34 influenza strain A. We determined influenza matrix gene expression of virus infected lungs using Q-PCR, and observed a decline in lung virus titres of influenza challenged mice vaccinated with the known HLA-A*02:01 binding immunogenic M(58–66) peptide [21], but no effect in mice immunized with a mixture of the four HLA double binding peptides.

### A case for T cell epitope-based anti-influenza A vaccine

The primary port of entry of the influenza virus is the mucosa of the respiratory tract. The adaptive immune system can provide immune protection against such mucosal pathogens through secretory IgA and IgM immunoglobulins. These can effectively prevent the virus from infecting its target cells, and the influenza viruses constantly go through antigenic changes to escape such neutralizing immunoglobulins. Thus, although a vaccine strategy aimed at generating humoral immunity can be very effective, it may not be possible to pursue it before the exact target is known.

Another arm of the adaptive immune system, that of HLA class I restricted, CD8 positive, cytotoxic T cells (CTL), specifically survey the internal metabolism of our cells and detect intracellular pathogens such as viruses Although this does not prevent the influenza virus from infecting the mucosal target cells, it does allow for a cytotoxic immune response, which can abort the transmission of infectious influenza virus from one host cell to the next thereby aiding in the recovery from the infection. The presence of HLA class I and II restricted epitope by the same antigenic peptide may add on additional anti-viral immunity by stimulating cross-protective IL-4-stimulated humoral responses and provide immunological memory. Thus combining bioinformatics, biochemistry, immunization technology and *in vivo* model systems might lead to generation of peptide-based vaccines capable of inducing cellular immunity towards upcoming influenza A infections.

In conclusion, HLA class I and II double binding small immunogenic peptides exist. By immunization, they simultaneously stimulate both HLA-I and–II restricted immune responses against the same peptide epitope. Thus double restricted peptides could be new candidates for peptide- based vaccines.

## Supporting Information

S1 Fig Flow cytometry–gating and IFNγproduction from CD8 and CD4 T cells. (TIF)

S2 Fig Representative dot plots showing CD4^+^ and CD8^+^ T cell depletion. (TIF)
