# Supplementary figures and images for: Immunogenicity of HLA Class I and II Double Restricted Influenza A-Derived Peptides
Source: PLoS One. 2016 Jan 5;11(1):e0145629. doi: 10.1371/journal.pone.0145629 (PMC4701504; doi:10.1371/journal.pone.0145629)

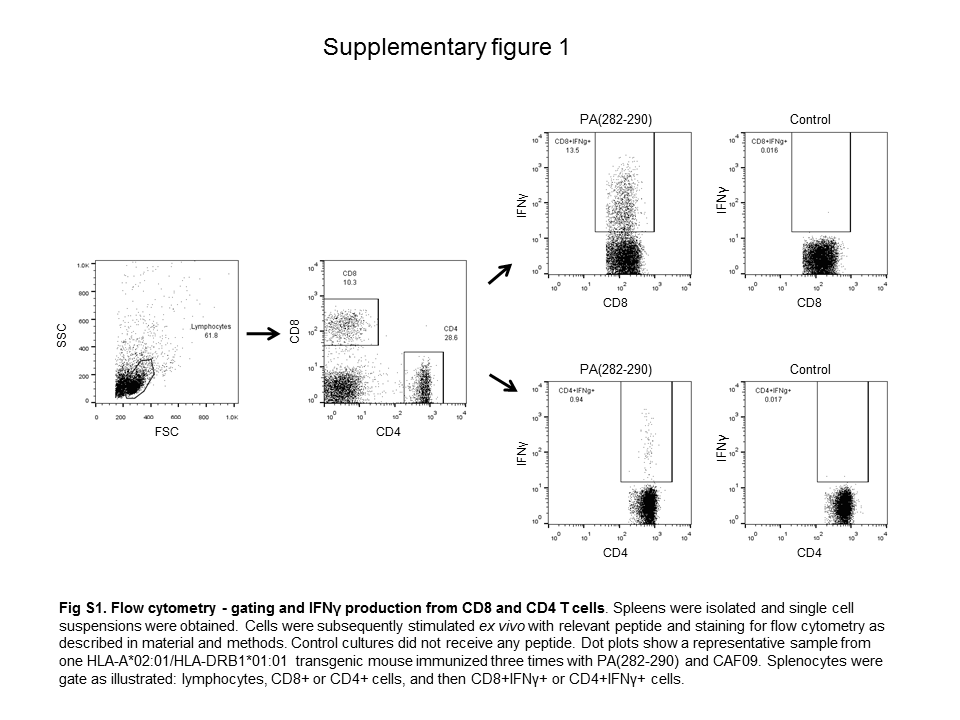

Supplement: S1 Fig — (TIF) [file pone.0145629.s001.tif]

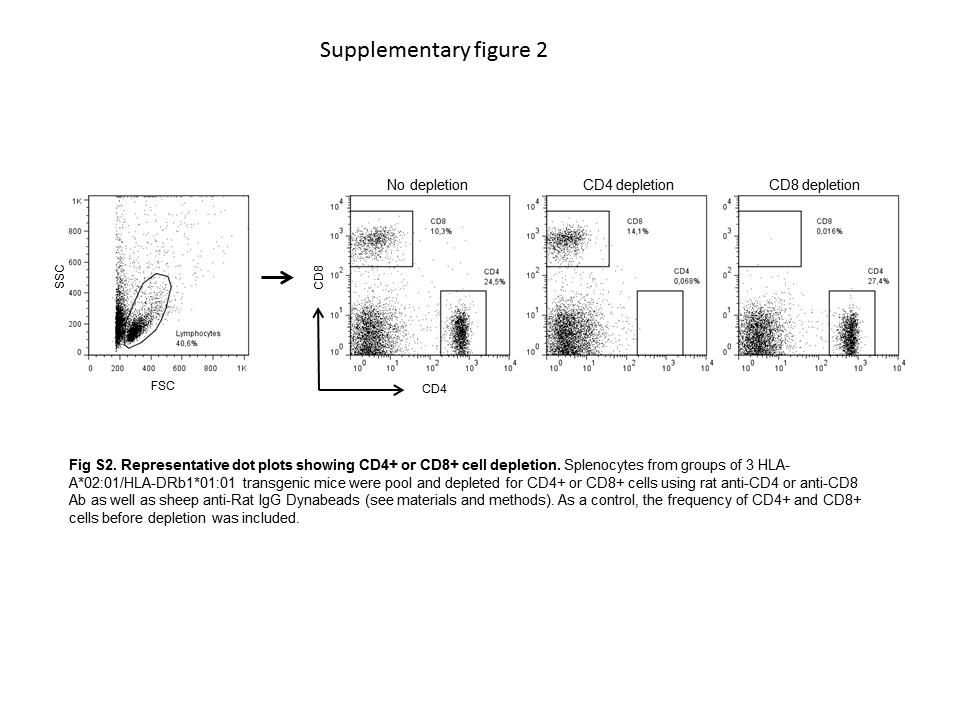

Supplement: S2 Fig — (TIF) [file pone.0145629.s002.tif]
